# Genomic-based genotype and drug susceptibility profile of *Mycobacterium kansasii* in China

**DOI:** 10.3389/fmicb.2025.1573448

**Published:** 2025-04-29

**Authors:** Yiting Wang, Xichao Ou, Bing Zhao, Hui Xia, Yang Zheng, Yang Zhou, Ruida Xing, Yuanyuan Song, Shengfen Wang, Yanlin Zhao, Huiwen Zheng

**Affiliations:** ^1^National Tuberculosis Reference Laboratory, Chinese Center for Disease Control and Prevention, Beijing, China; ^2^Beijing Center for Disease Control and Prevention, Beijing Institute of Tuberculosis Control, Institute for Immunization and Prevention, Beijing Academy for Preventive Medicine, Beijing, China; ^3^Laboratory of Respiratory Diseases, Beijing Key Laboratory of Pediatric Respiratory Infection Diseases, Beijing Children’s Hospital, Key Laboratory of Major Diseases in Children, Ministry of Education, National Clinical Research Center for Respiratory Diseases, National Center for Children’s Health, Beijing Pediatric Research Institute, Capital Medical University, Beijing, China

**Keywords:** *Mycobacterium kansasii*, drug susceptibility testing, genotype, whole genome sequencing (WGS), phylogenetic analysis

## Abstract

To analyze subtypes, microbiological characteristics and antimicrobial susceptibility of *Mycobacterium kansasii* in China, a total of 153 *M. kansasii* isolates, collected from national drug resistance surveillance, were genotyped with whole genome sequencing and explored the antimicrobial susceptibility with broth microdilution. All isolates were classified as *M. kansasii* type I based on Average Nucleotide Identity(ANI). The 153 *M. kansasii* representatives were differentiated into 3 clusters with 141 genotypes, including 17 isolates from a cluster and 136 isolates with unique patterns. The EXS-1, EXS-3 and EXS-5 regions were involved in all isolates. Rifabutin and clarithromycin were the most highly active against *M. kansasii* strains, with the susceptible rate of 100 and 99.35%, respectively. Followed by amikacin and linezolid, the resistance rate was 5.88 and 7.19%, respectively. The resistance rate to rifampin (RIF) was 22.22%. As for the antibiotics without the breakpoint values, all isolates had very low MIC_50_ (0.03 μg/mL) and MIC_90_ (≤0.06 μg/mL) values against bedaquiline, sutezolid, delamanid, and clofazimine. Except for ciprofloxacin and moxifloxacin, the resistance rate of other drugs in cluster 3 was higher than that in cluster 1 and cluster 2. In conclusion, *M. kansasii* type I was the predominant genotype in China, and rifabutin and clarithromycin presented strong activities. The new drugs, used for the treatment of multidrug - resistant tuberculosis, have the potential to be potent agents in the treatment of *M. kansasii* infection. The clustering might contribute to the high resistance rate of *M. kansasii*.

## Introduction

*Mycobacterium kansasii*, a leading cause of pulmonary, extrapulmonary and disseminated diseases in immunocompromised individuals, stands out as one of the most frequently isolated non-tuberculous mycobacterial (NTM) species worldwide (Hoefsloot et al., 2013; Campo and Campo, 1997; Pintado et al., 1999). Initially, seven subtypes (I-VII) along with two intermediate (I/II) and atypical (IIb) types, were taxonomically reclassified as species-level members of the *M. kansasii* complex (MKC) (Taillard et al., 2003; Iwamoto and Saito, 2006; Tagini et al., 2019; Jagielski et al., 2020). Though multiple molecular techniques have been developed to genotype *M. kansasii*, the resolution was low when relying solely on certain gene sequences (Zhang et al., 2004). Therefore, we conducted whole genome sequence (WGS) to enable high-resolution subtyping analysis.

Due to the difference in the pathogenicity of *M. kansasii* subtypes, it is necessary to perform drug susceptibility testing (DRS) to predict the clinical outcome of patients infected with *M. kansasii* (Kim et al., 2021; Li et al., 2016). Daily therapy with isoniazid (INH), rifampin (RIF), and ethambutol (EMB) was recommended by the American Thoracic Society/Infectious Disease Society of America (ATS/IDSA) for *M. kansasii* (Griffith et al., 2007). Given that treatment failure is associated with RIF resistance, there is an urgent need to conduct DRS for other medications (Griffith et al., 2007). Besides, considering the natural resistance to most antibacterial drugs for NTM, novel and more effective antibiotics were urgently needed (Raju et al., 2016). Whereas data on DRS of *M. kansasii* was limited. Therefore, this study was designed to analyze subtypes, microbiological characteristics and antimicrobial susceptibility of *M. kansasii* isolates in China.

## Materials and methods

### *M. kansasii* isolates and genotyping

Sputum specimens were collected from suspected pulmonary tuberculosis patients with acid-fast bacilli-positive isolates, from 8 provinces and 3 municipalities included in the Chinese Drug Resistance Surveillance Program (DRS) running between 2016 and 2020. Patients were excluded if the samples were unqualified. Clinical samples treated with NALC-NaOH were subsequently cultured on differential medium containing paranitrobenzoic acid (PNB) and thiophen-2-carboxylic acid hydrazide (TCH) to distinguish NTM from *Mycobacterium tuberculosis* complex (MTBC). Isolates identified as NTM using biochemical method were further identified as MKC by sequencing the genes encoding 16S rRNA, *hsp65*, *rpoB*, and the 16S–23S rRNA internal transcribed spacer (ITS), and non-purified isolates were excluded from further analysis. The MKC strains were further re-identified by MALDI-TOF-MS, representing I-VII genotypes of *Mycobacterium kansasii* spectra reference database, followed by WGS, which resulted in the final identification of all isolates as *M. kansasii*.

### Genome sequencing

All genomic DNA was extracted using the cetyl-trimethyl-ammonium bromide (CTAB) method, as previously described (Kigozi et al., 2018). Extracted genomic DNA was quantified using a Qubit 2.0 Fluorometer (Thermo Fisher, Singapore). Genomic DNA libraries were constructed using the Illumina Nextera kit according to the manufacturer’s protocol. Each library was sequenced using an Illumina NovaSeq 6,000 system (Illumina, Inc.) that generates 150-bp paired-reads from each end (read length of 2 × 150 bp) for each DNA fragment in a library. In order to secure exhaustive coverage of the target genome, each sample underwent sequencing to reach a depth of 150x and the genome coverage was requested to be above 90%. All whole-genome sequencing procedures were performed by Annoroad Gene Technology (Beijing, China).

The Raw Data were filtered to remove contaminants and low - quality reads before analysis. SPAdes (v3.15.5) and Unicycler (v0.5.0) were used for assembly in three steps: correction, assembly and mismatch correction. GapFiller (v1.11) optimized the assembly and filled gaps. Seqkit (v2.4.0) filtered out fragments < 200 bp for the final result. Prokka (v1.14.5) was used for annotation.

### Identifying

Resulting genomic sequences were analyzed to identify distinct subspecies according to average nucleotide identity (ANI) values, which were calculated using pyani.^1^ The results were visualized as a heatmap using R package *ComplexHeatmap* (Gu et al., 2016). Isolates yielding genome sequences with ANI values of >95% were deemed members of the same species (Kim et al., 2014). The utilization of ANI values for species - level discrimination can significantly enhance the resolution of identification, enabling more precise differentiation at the subspecies level.

### Phylogenetic tree construction

Core single nucleotide polymorphisms (SNPs) in genome sequences obtained for each isolate were detected by mapping reads to the *M. kansasii* reference strain (ATCC12478) sequence using Snippy v4.4.5, then core SNPs were concatenated and aligned using the snippy-multi script. A maximum likelihood tree was generated based on core-SNP alignment then the resulting tree was visualized using the Interactive Tree of Life (iTOL) online tool (Minh et al., 2020; Letunic and Bork, 2011).

### Virulence identification

Contigs were screened to detect known virulence genes using ABRicate and the Virulence Factors Database (VFDB). Next, virulence factor analysis was performed on potential virulence gene sequences with sequence coverage rates of ≥85% and rates of sequence identity with known virulence genes of ≥85% (Guo et al., 2022). Heatmaps were generated using the heatmap function of the R package.

### Antimycobacterial drug susceptibility testing

Antimicrobial susceptibility testing was performed on Sensititre SLOMYCO^®^ plates (Thermo Fisher Scientific, United States) according to Clinical and Laboratory Standards Institute (CLSI) guidelines (Parrish, 2023). Minimal inhibitory concentrations (MICs) determined within the concentration range of 0.0625–128 mg/mL were performed for a total of 17 antimicrobial agents (Table 1). The MIC is defined as the lowest concentration of antibiotic that inhibits visible mycobacterial growth. In this work, MIC test results were interpreted based on CLSI-recommended breakpoints (Parrish, 2023). The abovementioned *M. kansasii* reference strain was tested along with each batch of isolates as a quality control measure. Drug concentrations that inhibited the growth of tested isolates by 50 and 90% were expressed as MIC_50_ and MIC_90_ values, respectively.

**Table 1:** The MIC values and resistant rate of antibiotic for *M. kansasii.*

Antibiotics	MIC (μg/mL)					Number of resistant isolates (%)
	Sensitive	Intermediate	Resistant	MIC_50_	MIC_90_	
Rifabutin	≤2	–	≥4	0.25	0.5	0(0.00)
Clarithromycin	≤8	16	≥32	0.25	2	1(0.65)
Amikacin	≤16	32	≥64	4	16	9(5.88)
Linezolid	≤8	16	≥32	4	16	11(7.19)
Ciprofloxacin	≤1	2	≥4	1	4	19(12.42)
Moxifloxacin	≤1	2	≥4	0.25	8	32(20.92)
Rifampicin	≤1	–	≥2	0.5	8	34(22.22)
Trimethoprim/sulfamethoxazole	≤2/38	–	≥4/76	2	8	68(44.44)
Doxycycline	≤1	2–4	≥8	4	16	73(47.71)
Ethambutol	–	–	–	8	16	–
Isoniazid	–	–	–	2	8	–
Streptomycin	–	–	–	4	16	–
Ethionamide	–	–	–	0.6	2.5	–
Bedaquiline	–	–	–	0.03	0.03	–
Sutezolid	–	–	–	0.03	0.06	–
Delamanid	–	–	–	0.03	0.06	–
Clofazimin	–	–	–	0.03	0.03	–

MIC_50_: the minimum concentration of an antimicrobial agent required to inhibit the growth of 50% of the organisms. MIC_90_: the minimum concentration of an antimicrobial agent required to inhibit the growth of 90% of the organisms.

### Statistical analysis

All statistical analyses were performed using SPSS software, version 24 (IBM, United States). Intergroup difference was deemed statistically significant for results with *p* < 0.05. Drug MIC distributions for the 153 isolates were analyzed using GraphPad Prism software (version 7.00, La Jolla, CA, United States).

#### Sequence information

The WGS data has been submitted to the National Center for Biotechnology Information (NCBI) with the Bio project number of PRJNA925290 or submission ID: SUB12542119.

## Results

### Identification and genotyping

**Figure 1:**
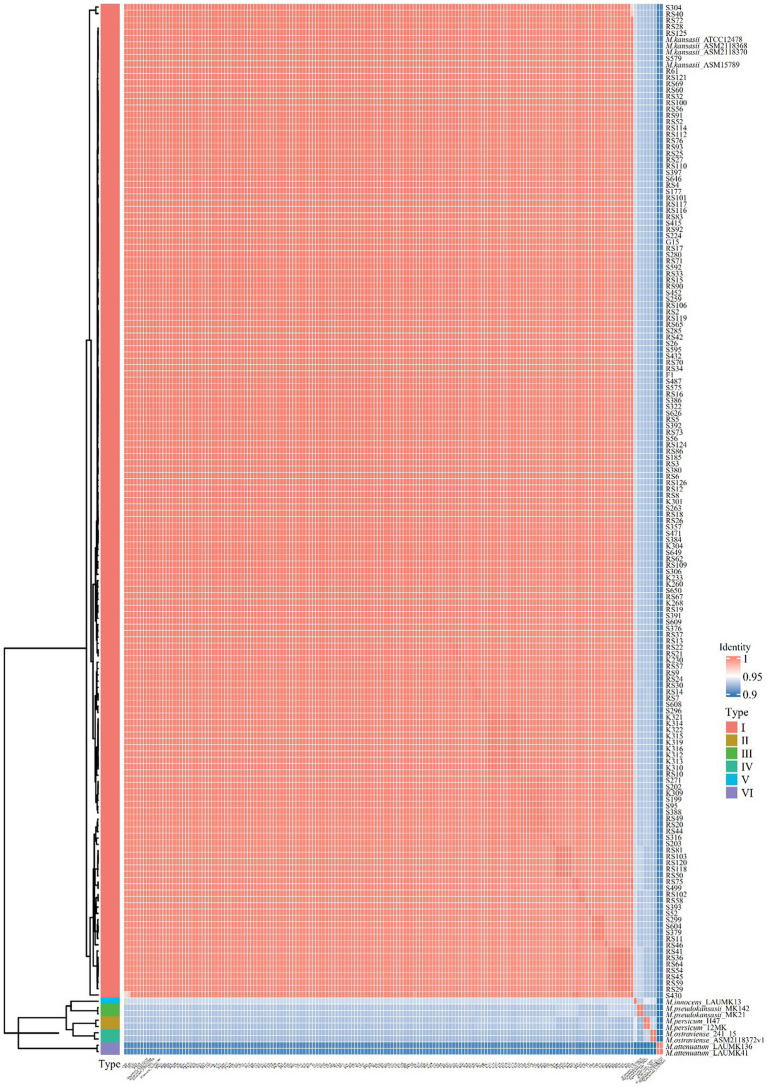
Pairwise comparisons of ANIs of *M. kansasii* subtypes I-VI. The heatmap displayed the Average Nucleotide Identity (ANI) values among 153 *M. kansasii* strains and the standard MKC strains. The color gradients in the heatmap represent different intensity levels of values. Red areas indicate higher ANI values, while blue areas signify lower ANI values. The bar chart on the left corresponds to different MKC species and conducts a clustering analysis of the samples.

**Figure 2:**
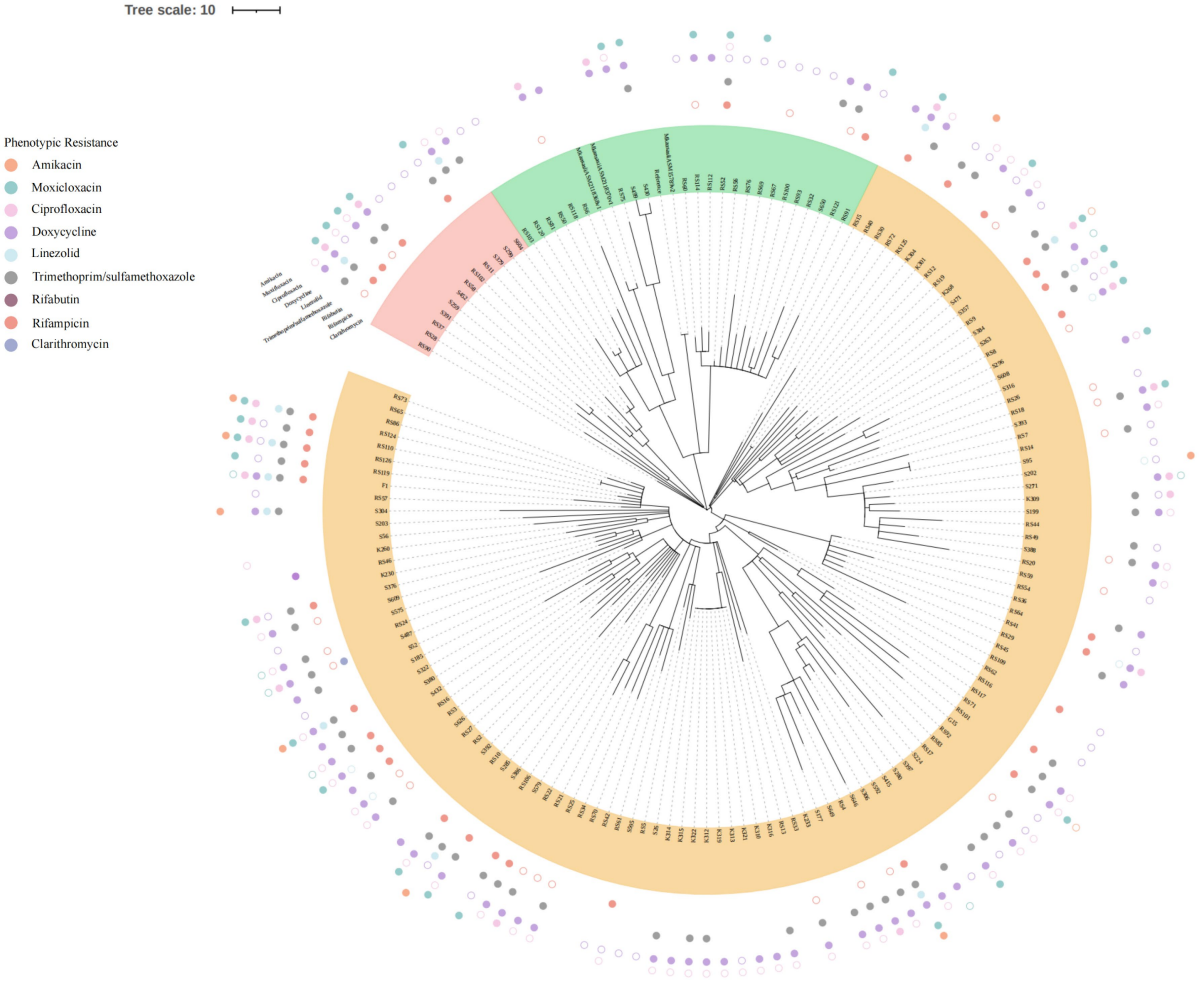
Phylogenetic tree based on core SNP. All 153MKC strains were divided into three clusters, with each cluster represented by a unique color. The outlying circles depicted the outcomes of the drug sensitivity tests. Distinct colors were employed to signify various drugs. Solid circles denoted drug resistance, while hollow circles represented intermedia.

All 153 isolates were ultimately classified as *M. kansasii* based on ANI values, which indicated that the genome sequence of each isolate shared >95% identity with that of the *M. kansasii* reference strain ATCC 12478 (Figure 1). The 153 *M. kansasii* representatives were differentiated into 3 clusters with 141 genotypes, including 17 isolates from a cluster (2–9 isolates per cluster) and 136 isolates with unique patterns (Figure 2). The clustering rate of *M. kansasii* was 11.1% (17/153).

### Virulence factor-encoding gene

**Figure 3:**
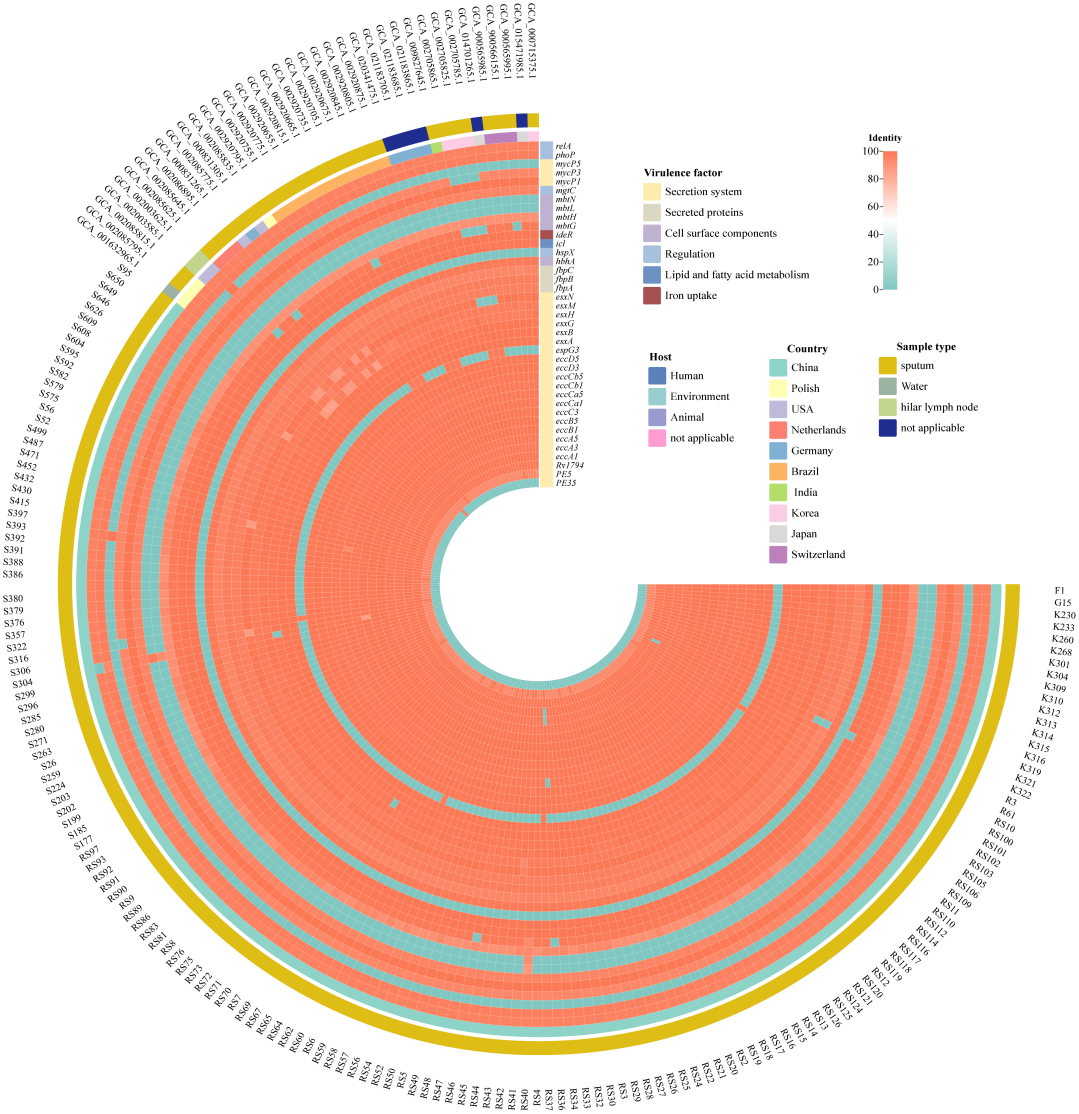
Virulence factors in *M. kansasii* from different sources. The heatmap displayed the identity of multiple virulence genes. The horizontal axis is labeled with samples while the vertical axis enumerates individual virulence genes. A color—coded gradient is employed to depict the degree of identity among the genes. A score of 100 is represented by red, signifying a high level of sequence similarity, conversely, a light blue color corresponds to an identity score of 0, indicating a lack of significant similarity.

Referring to the filter threshold of the reference virulence gene, homologous genes with a coverage of 80% and a similarity of 80% were listed in Figure 3. The EXS-1, EXS-3 and EXS-5 regions were involved in all isolates. And *fbpA*, *fbpB*, *fbpC*, *hbhA*, *ideR*, *mbtH*, *mgtC*, and *phoP* gene were present in all *M. kansasii* subtype I strains.

### Drug susceptibility testing

**Figure 4:**
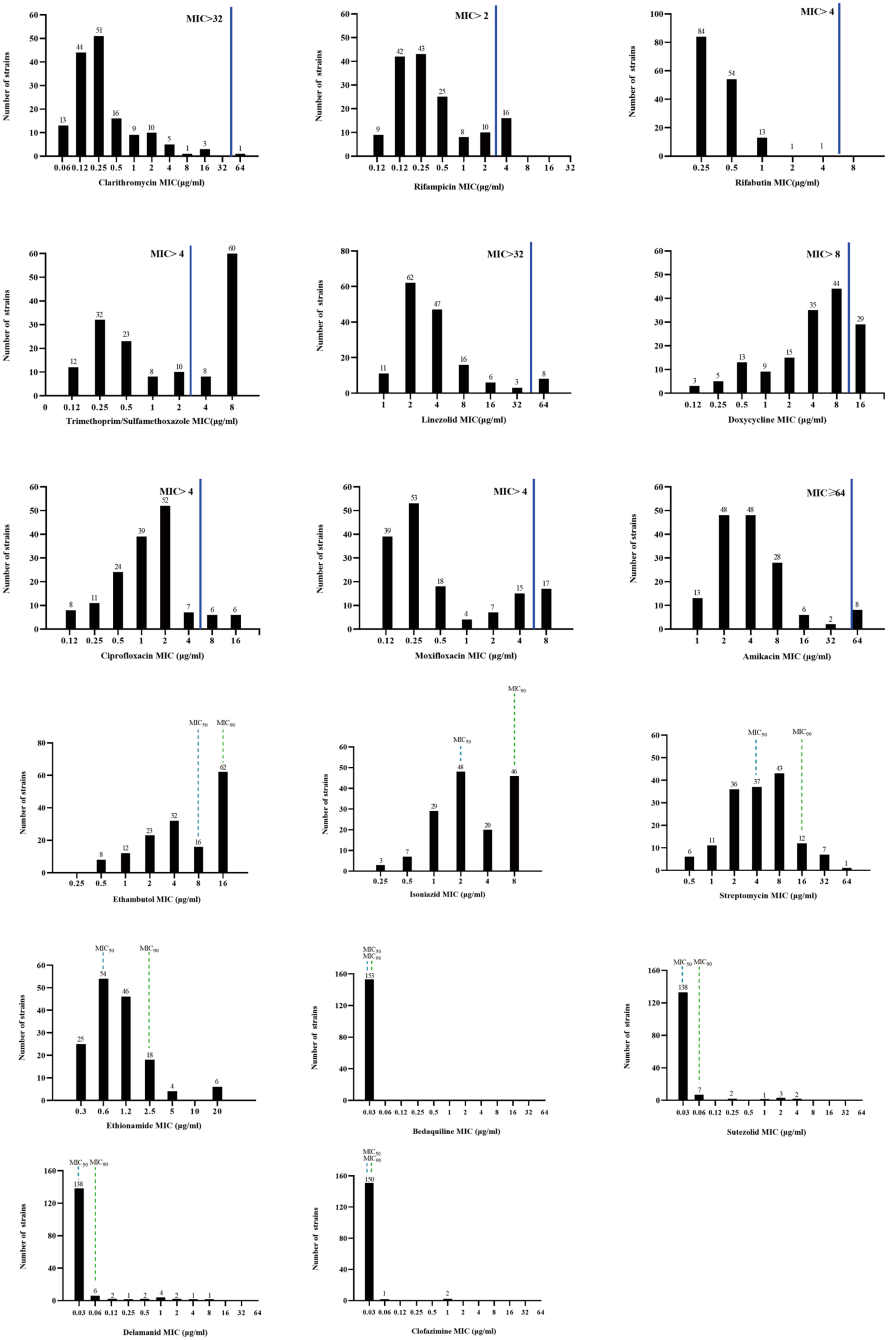
MIC distributions of *M. kansasii* clinical isolates. The solid blue line indicates the CLSI-recommended threshold for determining drug susceptibility, above which a resistant strain is considered. Drugs lacking critical values of MIC50 and MIC90 are denoted by dashed lines. MIC_50_: the minimum concentration of an antimicrobial agent required to inhibit the growth of 50% of the organisms. MIC_90_: the minimum concentration of an antimicrobial agent required to inhibit the growth of 90% of the organisms.

For the antimicrobial agents with the breakpoint values recommended by CLSI, rifabutin (RFB) and clarithromycin (CLA) were the most highly active against *M. kansasii* strains, with the susceptibility rate of 100% and 99.35%, respectively. Followed by amikacin (AMK) and linezolid (LZD), the resistance rate was 5.88% and 7.19%, respectively (Table 1). For the fluoroquinolones, the resistance rate to ciprofloxacin (CIP) and moxifloxacin (MXF) was 12.42% and 20.92%, respectively. Of the 22 isolates resistant to MXF, 10 isolates (45.45%) exhibited resistance to CIP (Supplementary Figure S1). The resistance rate to RIF was 22.22%. As for the antibiotics without breakpoint values, all isolates had very low MIC_50_ (0.03 μg/mL) and MIC_90_ (≤0.06 μg/mL) values against bedaquiline (BDQ), sutezolid (SZD), delamanid (DLM), and clofazimin (CFZ). Ethionamide (ETO) was also active against *M. kansasii*, with MIC_50_ and MIC_90_ values of 0.6 μg/mL and 2.5 μg/mL, respectively (Figure 4).

### Association between drug resistance profiles and genotypes

Although no statistically significant associations between clusters and drug resistance profiles were observed, CLA, RIF, RFB, TMP/SMX, LZD, DOX, and AMK in cluster 3 were 1.18, 24.71, 1.18, 49.41, 8.24, 50.59, and 8.24% respectively, which exceeded corresponding rates observed for cluster 1 (0, 20, 0, 32.5, 7.5, 45, and 2.5%) and cluster 2 (0, 17.86, 0, 42.86, 0, 46.43, and 3.57%) isolates. Meanwhile, CIP and MXF resistance rates of cluster 3 (10.59 and 15.29%) isolates were lower than corresponding rates obtained for cluster 1 (12.50 and 30%) and cluster 2 (14.29 and 25%) isolates (Table 2).

**Table 2:** Drug resistance profiles among different *M. kansasii* genotypes.

Drug		No. susceptible (%)	No. resistant (%)	*X* ^2^	*p*-value
Clarithromycin	Cluster1	40(100.00)	0(0.00)	0.80	0.67
	Cluster2	27(96.43)	0(0.00)		
	Cluster3	84(98.82)	1(1.18)		
Rifampicin	Cluster1	32(80.00)	8(20.00)	0.73	0.70
	Cluster2	23(82.14)	5(17.86)		
	Cluster3	64(75.29)	21(24.71)		
Rifabutin	Cluster1	40(100.00)	0(0.00)	0.81	0.67
	Cluster2	28(100.00)	0(0.00)		
	Cluster3	84(98.82)	1(1.18)		
Trimethoprim/sulfamethoxazole	Cluster1	27(67.50)	13(32.5)	3.17	0.21
	Cluster2	16(57.14)	12(42.86)		
	Cluster3	43(50.59)	42(49.41)		
Linezolid	Cluster1	37(92.50)	3(7.50)	2.24	0.33
	Cluster2	25(89.29)	0(0.00)		
	Cluster3	75(88.24)	7(8.24)		
Doxycycline	Cluster1	4(10.00)	18(45.00)	1.49	0.48
	Cluster2	5(17.86)	13(46.43)		
	Cluster3	20(23.53)	43(50.59)		
Ciprofloxacin	Cluster1	27(0.68)	5(12.50)	0.69	0.71
	Cluster2	12(42.86)	4(14.29)		
	Cluster3	44(51.76)	9(10.59)		
Moxifloxacin	Cluster1	28(70.00)	12(30.00)	3.41	0.18
	Cluster2	19(67.86)	7(25.00)		
	Cluster3	67(78.82)	13(15.29)		
Amikacin	Cluster1	39(97.50)	1(2.50)	2.00	0.37
	Cluster2	27(96.43)	1(3.57)		
	Cluster3	77(90.59)	7(8.24)		

*P < 0.05 was defined to be statistically significant.*

## Discussion

*M. kansasii*, the most pathogenic subspecies, is responsible for most NTM infections worldwide (Luo et al., 2021; Johnston et al., 2017). Previous studies in China have reported *M. kansasii* as predominant species, comprising 71.8% of MKC isolates, with 14.1% belonging to *M. persicum* (Li et al., 2016). In contrast, all MKC isolates analyzed in this study were classified as *M. kansasii.* The discrepancy may be attributed to the fact that *M. persicum* is an opportunistic pathogen, which primarily infects immunocompromised individuals, and thus was not detected in our cohort of immunocompetent patients (Taillard et al., 2003). Alternatively, *M. kansasii* isolates in this study may exhibit greater genetic homogeneity compared to those circulating in other regions of China (Mokrousov, 2017).

Intriguingly, the clustering rate observed in the current study (11%) was substantially lower than those reported in prior investigations (68–99.3%) (Taillard et al., 2003; Zhang et al., 2004; Bakula et al., 2018). This discrepancy may be attributed to methodological differences in typing methods used across studies. Earlier studies predominantly utilized variable number tandem repeat (VNTR)-based assays or pulsed-field gel electrophoresis (PFGE) for MKC typing (Taillard et al., 2003; Zhang et al., 2004; Bakula et al., 2018). However, compared to WGS, these conventional methods are more time-consuming, labor-intensive, resource-intensive, require a higher degree of expertise, and offer significantly lower discriminatory power, highlighting the superior of WGS as a high-throughput and high-resolution genotyping platform (Bakula et al., 2018).

The type-VII secretion system comprises five homologous secretion systems (ESX-1 to ESX-5), which are critical for the pathogenicity of both *M. tuberculosis* and *M. kansasii* (Guan et al., 2020). In this study, genes encoding the conserved components of the EXS-1, EXS-3, and EXS-5 system were detected in genomes of all 153 *M. kansasii* isolates. Additionally, *esxG* gene, which encodes an ESX-3 substrate known to enhance *M. tuberculosis* virulence by facilitating iron acquisition (Tufariello et al., 2016), was also universally present. Moreover, the *fbpC* gene, encoding an enzyme involved in transferring mycolic acid from trehalose monomycolate to the mycolyl-arabinogalactan complex (Favrot et al., 2013), was detected in 99.35% (152/153) of isolates. By contrast, results of another study conducted in China by Guo et al., revealed that the *fbpC* gene was absent in genomes of all *M. kansasii* isolates, while the *esxG* gene was detected in only 6.7% (4/60) of cases (Guo et al., 2022). We hypothesize that these discrepancies may reflect variations in the virulence profiles of *M. kansasii* strains circulating in different geographic areas.

Core treatment regimens for *M. kansasii* infections consist of RIF, INH, and EMB (Parrish, 2023). Nevertheless, the clinical utility of INH and EMB is limited, as their MIC values correlate poorly with treatment outcomes, rendering them unreliable prognostic indicators (Parrish, 2023). In this study, the RIF resistance rate was 22.22% (34/153), similar to that reported in Shanghai (20.0%) (Guo et al., 2022), but lower than that observed in Beijing, China (56.4%) (Li et al., 2016). Notably, our resistance rates were higher than those documented in Poland and Brazil, where all tested isolates remained fully susceptible to RIF (Bakuła et al., 2018; de Carvalho et al., 2019). These disparities suggest potential geographical variations in resistance patterns, possibly influenced by regional differences in antibiotic usage and susceptibility profiles (Bakuła et al., 2018). In cases of treatment failure associated with RIF resistance, DST for secondary antimicrobial agents should be conducted to identify effective alternatives and optimize therapeutic outcomes (Parrish, 2023). Consistent with previous findings, all isolates in this study remained susceptible to RFB, reinforcing its potential as an effective substitute for RIF in clinical practice (Guo et al., 2022).

The CLA susceptibility rate for our isolates was 99.35% (152/153), aligning with previous reports of <1% resistance (Guo et al., 2022; de Carvalho et al., 2019). However, another study reported a substantially higher CLA resistance rate (20.5%), possibly due to macrolide overuse driving resistance development (Li et al., 2016). Additionally, AMK and LZD exhibited excellent *in vitro* activities against *M. kansasii,* with sensitivity rates exceeding 90%, indicating them to be promising options for *M. kansasii* infections (Guo et al., 2022; de Carvalho et al., 2019). SZD, a newer oxazolidinone, also exhibited excellent in vitro activity against *M. kansasii*, consistent with previously reported (Yu et al., 2021; Zheng et al., 2023). Notably, BDQ, DLM, and CFZ, which are newer drugs used to treat multidrug-resistant tuberculosis cases, demonstrated consistent activity against *M. kansasii* in our study and previous research (Zheng et al., 2022), highlighting their potential as alternative therapies for *M. kansasii* infections.

This research has several limitations. First, as this retrospective study relied on tuberculosis DRS, the clinical information and treatment history were unavailable for patients diagnosed with *M. kansasii* pulmonary disease, who were discontinued from follow-up. Second, we did not collect MKC isolates from environmental sources to demonstrate the sources of clinical infections. Third, all *M. kansasii* isolates were obtained from patients in China and thus the conclusions drawn from an analysis of these isolates may not be generalizable to clinical isolates from other countries.

## Conclusion

*M. kansasii* type I was the predominant genotype in China, and RFB and CLR presented strong activities against *M. kansasii* strains. Three clusters were separated based on core SNP throughout the genome. The new drugs BDQ, DLM, SZD and CFZ have the potential to be potent agents for the treatment of *M. kansasii* infection.

## Data Availability

The datasets presented in this study can be found in online repositories. The names of the repository/repositories and accession number(s) can be found in the article/Supplementary material.

## Footnotes

^1^ https://github.com/widdowquinn/pyani
