# Supplementary figures and images for: Genomic-based genotype and drug susceptibility profile of Mycobacterium kansasii in China
Source: Front Microbiol. 2025 Apr 29;16:1573448. doi: 10.3389/fmicb.2025.1573448 (PMC12069364; doi:10.3389/fmicb.2025.1573448)

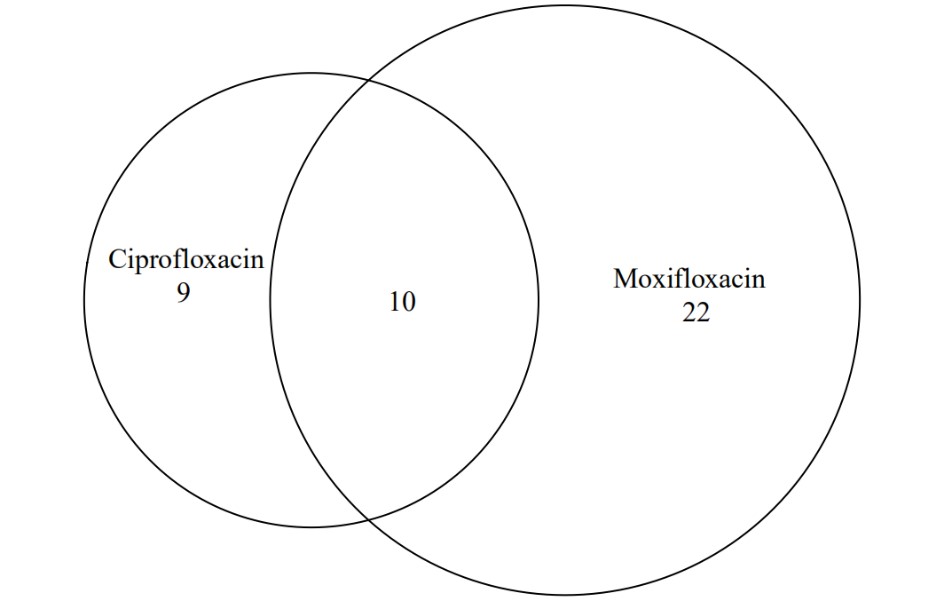

Supplement: Supplementary file 1 [file Image_1.jpg]
